# Endothelial Cells Obtained from Patients Affected by Chronic Venous Disease Exhibit a Pro-Inflammatory Phenotype

**DOI:** 10.1371/journal.pone.0039543

**Published:** 2012-06-21

**Authors:** Veronica Tisato, Giorgio Zauli, Rebecca Voltan, Sergio Gianesini, Maria Grazia di Iasio, Ilaria Volpi, Guido Fiorentini, Paolo Zamboni, Paola Secchiero

**Affiliations:** 1 Department of Morphology and Embryology and LTTA Centre, University of Ferrara, Ferrara, Italy; 2 Institute for Maternal and Child Health, IRCCS Burlo Garofolo, Trieste, Italy; 3 Vascular Disease Center, University of Ferrara, Ferrara, Italy; Beth Israel Deaconess Medical Center, United States of America

## Abstract

**Background:**

The inflammatory properties of vein endothelium in relation to chronic venous disease (CVD) have been poorly investigated. Therefore, new insights on the characteristics of large vein endothelium would increase our knowledge of large vessel physiopathology.

**Methodology/Principal Findings:**

Surgical specimens of veins were obtained from the tertiary venous network (R3) and/or saphenous vein (SF) of patients affected by CVD and from control individuals. Highly purified venous endothelial cell (VEC) cultures obtained from CVD patients were characterized for morphological, phenotypic and functional properties compared to control VEC. An increase of CD31/PECAM-1, CD146 and ICAM-1 surface levels was documented at flow cytometry in pathological VEC with respect to normal controls. Of note, the strongest expression of these pro-inflammatory markers was observed in VEC obtained from patients with more advanced disease. Similarly, spontaneous cell proliferation and resistance to starvation was higher in pathological than in normal VEC, while the migratory response of VEC showed an opposite trend, being significantly lower in VEC obtained from pathological specimens. In addition, in keeping with a higher baseline transcriptional activity of NF-kB, the release of the pro-inflammatory cytokines osteoprotegerin (OPG) and vascular endothelial growth factor (VEGF) was higher in pathological VEC cultures with respect to control VEC. Interestingly, there was a systemic correlation to these *in vitro* data, as demonstrated by higher serum OPG and VEGF levels in CVD patients with respect to normal healthy controls.

**Conclusion/Significance:**

Taken together, these data indicate that large vein endothelial cells obtained from CVD patients exhibit a pro-inflammatory phenotype, which might significantly contribute to systemic inflammation in CVD patients.

## Introduction

Chronic venous disease (CVD) is a common problem that has a significant impact on afflicted individuals and on the healthcare system. Normal venous function requires the axial veins with a series of venous valves, the perforating veins to allow communication of the superficial with the deep venous system, and the venous muscle pumps. The dysfunction of any of the normal structures may lead to the development of chronic venous insufficiency [1]. Venous outcomes assessment tools have been used to evaluate the severity of CVD, but little information is available on the correlation between the currently used classifications, such as the clinical signs, etiology, anatomic distribution and pathophysiology (CEAP), and the status of venous endothelial cells in the afflicted veins.

The vascular endothelium acts as a barrier between tissue and blood and regulates diverse functions, such as the local and central hemodynamic and the exchange of nutrients and metabolites [2]–[4]. Although it is recognized that the endothelium also plays important roles in inflammatory diseases, atherosclerosis and thrombosis [5]–[8], it should be emphasized that the endothelial monolayer is made up by a diverse family of cells that, depending on the location in the vascular tree, displays variable responses to a variety of stimuli. While the endothelial phenotype in arteries is characterized by the release of substances that influence vascular smooth muscle tone, vessel diameter, and local blood perfusion [9], the endothelium in post-capillary venules is specialized at responding to local inflammatory stimuli by increasing its own permeability toward macromolecules and by expressing molecules that mediate recruitment of leukocytes to the sites of inflammation [10]. Moreover, endothelial function is influenced by the developmental origin of the cells as well as by local blood flow dynamics [11], [12]. In contrast to the endothelium of arteries and venules, little is known about the endothelial function in large veins [13], although the responses of large vein endothelium play important roles in clinical disease and it has been proposed that destruction of venous valves leading to chronic venous congestion is imposed by inflammatory mechanisms [14].

On these bases, we sought to elucidate the physiopathological features of large vein endothelium at different stages of CVD. For this purpose, we have set up an efficient system of endothelial cells isolation from small pathological surgical specimens, which allowed us to characterize *ex-vivo* pathological VEC cultures for their phenotype, proliferation/survival, migration properties, release of the pro-inflammatory cytokines osteoprotegerin (OPG) [15] and vascular endothelial growth factor (VEGF), as well as for the levels of activation of the transcription factor NF-kB.

## Materials and Methods

### Recruitment of patients and samples collection

Fifty-four patients affected by primary CVD with superficial venous reflux were enrolled in this study and their main demographic/clinical characteristics are reported in **Table** **1**. The surgical specimens were collected during surgery for conservative and hemodynamic treatment of venous insufficiency in ambulatory care. Briefly, target of the procedure was to perform an effective treatment of CVD on ambulatory bases, sparing the saphenous vein, restoring the saphenous drainage and permitting a treatment of the recurrences by means of the same technique. Surgical pathological specimens of veins were collected from the tertiary venous network (referred as R3) and/or saphenous vein (referred as SF). As control, SF VEC (n = 5) were obtained from Promo-cell (Heidelberg, Germany). Serum samples were collected from primary CVD patients (n = 32) at the time of CEAP disease stage diagnosis and from sex- and age-matched healthy controls (n = 44). Venous blood samples from CVD patients and controls were immediately centrifuged at 3000 rpm for 15 minutes and serum was stored at −80°C in single-use aliquots. The procedures followed were in accordance with the Declaration of Helsinki, approved by the institutional review board (University-Hospital of Ferrara) and all participant subjects gave written informed consent.

**Table 1:** Baseline characteristics of subjects.

	n = 54	%
**Patient Age** (**years**)		
≤35	4	7%
36–50	13	24%
≥51	37	69%
**Gender**		
Men/Women	13/41	24%/76%
**Body mass index** (**kg/m2**)		
≤20	3	6%
21–32	47	87%
≥33	4	7%
**Blood Pressure** (**mmHg**)		
Hypertensive patients (total)	15	28%
Normalized under treatment	13	
Normotensive patients	39	72%
**Family history**		
Yes/No	43/11	80%/20%
**Relevant medical history**	25	46%
DM2	1	2%
Impaired fasting glycaemia	2	4%
Atherosclerosis	3	5%
Cardiac Disease	2	4%
Hypercholesterolaemia	5	9%
Current smoker	12	22%
**CEAP criteria**		
Clinical		
C2	13	24%
C3	35	65%
C4	5	9%
C5	1	2%
Etiology		
Primary	54	100%
Secondary	0	0%
Anatomy		
Deep venous reflux	0	0%
Superficial venous reflux	54	100%
Deep and superficial reflux	0	0%
Pathophysiology		
Reflux only	54	100%
Reflux and obstruction	0	0%

### Preparation of veins for scanning electron microscopy

After collection, venous segments intended for scanning electron microscopy (SEM) analysis were rapidly cut longitudinally, washed and placed in 2.5% glutaraldehyde for 24 hours at 4°C followed by 2 hours of incubation in 1% osmium tetroxide at room temperature. The samples were then treated with decreasing concentration of ethanol ending with a passage on propylene oxide. Finally, the samples were removed and covered with gold through sputter deposition (S 150 Sputter Coater Edwards, England) and examined under an AG-EVO®40 scanning electron microscope (Cambridge, England).

### VEC isolation and culture

The surgical fragments were removed from the collection pot, placed in a sterile Petri dish and all visible fat and connective tissues were removed by scalpel. The tissue was opened longitudinally in order to expose the endothelium and washed twice in PBS (Sigma Chemical Co, St Louis, MO). A 0.05% collagenase type 1 solution (Worthington, Lakewood, NJ) was added into a new Petri dish and the tissue was gently added on top of the solution in order to put the endothelium in direct contact with the enzyme solution. After 20 minutes of digestion at 37°C, the tissue was turned face up and the endothelium was gently scraped to detach endothelial cells. The containing-cells solution was collected in a sterile 50 ml tube and the enzyme activity was neutralized by adding fetal calf serum (Gibco BRL, Grand Island, NY) to a volume ratio of 1∶1. Cells were centrifuged and after two washes in PBS they were seeded in EGM2 medium (Lonza, Walkersville, MD) with 2% FBS and full supplements (EGM2 Bullet kit, Lonza) in multi-well plates (Corning Costar, Cambridge, MA) coated with fibronectin (BD, Becton Dickinson, San José, CA) at 5 μg/cm^2^, as previously described [16]. Cultures were placed in a 37°C humidified incubator at 5% CO_2_, washed after 72 hours and maintained under these conditions with medium changed every 3 days until they attained confluence and were designated P0 cultures. Cells were detached with trypsin-EDTA solution (Lonza) and sub-cultured to approximately 80% confluence for cell banking and to perform *in vitro* experiments. For this study, cells were used within passages 3 to 7.

### Cytofluorimetric characterization of primary VEC

The purity of primary VEC cultures was evaluated by multicolour flow cytometry analysis performed on a four lasers BD FACSAria™ II (BD Bioscience). Instrument parameters were set up and monitored as described below. In brief, BD™ Cytometer Setup & Tracking Beads were used to define the cytometer's initial baseline status (the voltage tolerance range). Once these baseline measurements were defined, the Cytometer Setup and Tracking Beads were subsequently used to run day-to-day cytometer performance checks. Experimental voltages were established by using BD™ CompBeads labelled with the antibodies used for the experiments. Cells were detached with trypsin-EDTA, washed and 5×10^5^ cells were resuspended in 200 μl of PBS containing 1% BSA (Sigma-Aldrich) and incubated 30 minutes at 4°C with the following anti-human endothelial-defining antibodies (Ab): FITC-conjugated anti-CD146 (Miltenyi Biotec, Gladbach, Germany, Clone 541-10B2), Horizon V450-A-conjugated anti-CD144 (BD, Clone 55-7H1), PE-A-conjugated anti-CD31 (Miltenyi Biotec, Clone AC128), Alexa Fluor 647-A-conjugated anti-CD105 (BD, Clone 266), PE-Cy7-A-conjugated anti-CD34 (BD, Clone 581), APC-H7-A-conjugated anti-CD45 (BD, Clone 2D1) and Horizon v500-A-conjugated anti-CD14 (BD, Clone M5E2). Data files were collected and analysed using the FACSDiva software program (version 6.1.3; BD) and displayed as single histogram colours and two-colours dot plots to measure the proportion of the single-positive or double-positive cells. Only cell cultures characterized by endothelial cell purity greater than 90% (n = 21), as evaluated by multicolour flow cytometry analysis, were used for subsequent studies. At different *in vitro* passages, VEC cultures were analysed by flow cytometry using the following Abs: FITC-conjugated anti-CD146 (BD), PE-A-conjugated anti-CD31 (BD), PE-A-conjugated anti-CD105 (BD, clone 266), FITC-conjugated anti-ICAM-1 (R&D, Minneapolis-MN, Clone BBIG-I1) and FITC-conjugated anti-VCAM (R&D, Clone BBIG-V3). Non-specific fluorescence was assessed by incubation with isotype-matched conjugated mAbs. In some experiments, the degree of apoptosis on detached endothelial cells was analysed by propidium iodide/annexin-V staining performed as previously described [17], [18].

### Proliferation and migration assays

Cell proliferation and migration were performed using the DP version of the xCELLigence real time cell analyzer RTCA (Roche Diagnostics, Mannheim, Germany), which records changes in impedance (reported as a cell index-CI) over a prolonged time course in a non-invasive system. All data were then analysed using the xCELLigence software (Roche, version 1.2.1). Briefly, for the proliferation assay the background impedance of RTCA DP E-Plates 16 was performed using the standard protocol provided in the software with 100 µL of EGM-2. Endothelial cells were seeded in quadruplicate at three different concentrations in fibronectin-pre-coated wells with 100 µL of complete (2% FBS and cytokines/growth factors) EGM2 and left to equilibrate at room temperature for 30 minutes. The CI of the proliferating cells was recorded up to 72 hours and data were expressed as mean of CI normalized to the CI recorded at 4 hours. For assays under starvation cells were allowed to adhere and proliferate in complete EGM2 for 24 hours before changing the medium with EBM2 without serum and cytokines/growth factors. The CI of the cells was recorded up to 72 hours after the beginning of the starvation and data were expressed as mean of CI normalized to the CI recorded at 24 hours.

Migration experiments were performed using fibronectin-pre-coated RTCA DP CIM-Plates 16. Cells were seeded in the upper chamber in quadruplicate at three different concentrations and left to equilibrate at room temperature for 30 minutes. Migration kinetics were analyzed in the absence or presence of 10% FBS in the bottom chamber and recorded up to 6 hours.

### Real-time reverse transcription-PCR analysis

Expression levels of ICAM-1 and VCAM were evaluated in VEC cultures either left untreated or treated with 2 ng/ml of TNF-α (R&D System) for 18 hours by quantitative RT-PCR. Total RNA was extracted from cells by using RNeasy Plus mini kit (Qiagen, Hilden, Germany) according to the supplier's instructions. The quality of the total RNA preparation was verified by agarose gel and, when necessary, further purification was performed with the RNeasy cleanup system (Qiagen) to remove chromatin DNA. Total RNA was transcribed into cDNA, using the QuantiTect Reverse Transcription kit (Qiagen). Analysis of ICAM-1 and VCAM gene expression was carried out using the SYBR Green real-time PCR detection method with the SABiosciences RT2 Real-Time^TM^ Gene expression assays, that include specific validated primer sets and PCR master mixes (SABiosciences, Frederick, MD), as previously described [19]. All samples were run in triplicate by using the real time thermal analyzer rotor-gene^TM^ 6000 (Corbett, Cambridge, UK). Expression values were normalized to the housekeeping gene POLR2A amplified in the same sample.

### Assay for NF-kB DNA binding

Baseline levels of active NF-kB were measured in protein cell extracts using the TransAM NF-kB p65 kit (Active Motif, Rixensart, Belgium), which evaluates the form of NF-kB able to bind specifically to an oligonucleotide containing the NF-kB consensus site (5′-GGGACTTTCC-3′) attached to a 96-well plate. Assays were performed in duplicates, according to the manufacturer's instructions. NF-kB DNA binding activity was determined as absorbance values measured by using an Anthos 2010 ELISA reader (Anthos Labtec Instruments Ges.m.b.H, Salzburg, Austria). Increase in fluorescence was linear over extract concentration.

### Enzyme-linked immunosorbent assay (ELISA) for OPG and VEGF measurement

Serum samples and endothelial culture supernatants were analysed for the pro-inflammatory cytokines OPG (Alexis Biochemicals, Lausen, Switzerland) and VEGF (R&D System) by using ELISA kits according to the manufacturer's instructions and as previously described [20]. Measurements were done in duplicates and the results were read at an optical density of 450 nm using an Anthos 2010 ELISA reader (Anthos Labtec Instruments Ges.m.b.H). Sensitivity of the OPG assay was 2.8 pg/ml, the intra- and inter-assay coefficients of variation (CV) were 9% and <10%, respectively. Sensitivity of the VEGF assay was 5 pg/ml, the intra- and inter-assay coefficients of variation (CV) were 7% and <9%, respectively.

### Statistical analysis

Descriptive statistical were calculated. For each set of experiments, values were reported either as means±SD or box plots were used to show the median, minimum and maximum values, and 25th to 75th percentiles. The results were evaluated by using Student's T and the Mann–Whitney rank-sum tests, when appropriate. Statistical significance was defined as p<0.05.

## Results

### *In situ* morphological characterization of large vein endothelium

**Figure 1:**
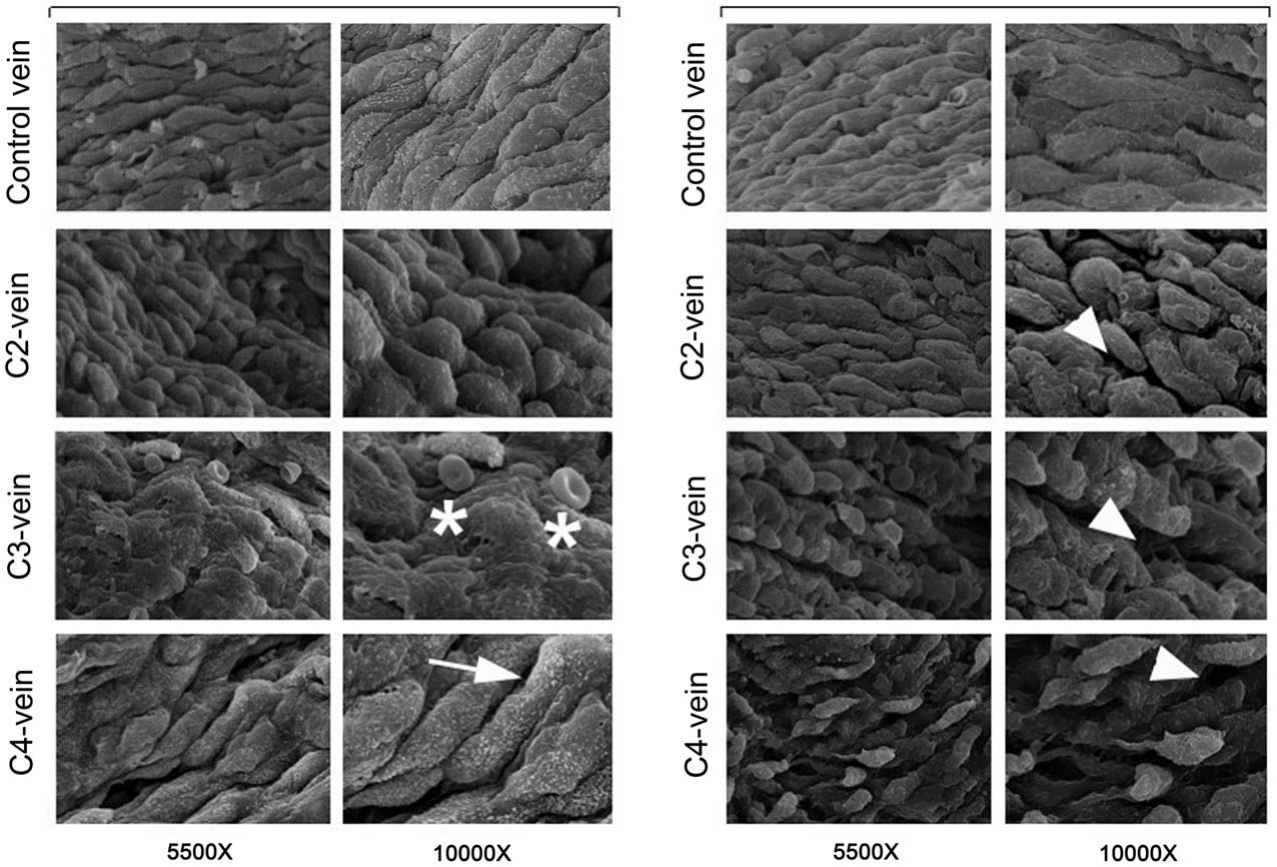
*In situ* morphological characterization of large vein endothelium. Morphological investigation of endothelium obtained from control and CVD (from C2 to C4 stages) veins was performed by SEM. A representative panel composed of images from 2 control specimens and 6 pathological veins (2 for each CEAP stage) is shown. In C2-, C3- and C4-veins, luminal pathological vessel surfaces were covered by endothelial cells characterized by an irregular orientation and several morphological abnormalities, such as: discontinuous surface of the endothelium (arrowheads), which increases with the severity of the disease, presence of plaques covering the endothelial surface (asterisks) and/or presence of numerous microvilli (arrow). Red cells adhering to endothelium were frequently observed (left C3 images).

In order to start to characterize the properties of venous endothelial cells obtained from patients affected by different stages of CVD, we have collected surgical specimens from 54 patients, with clinical signs of CVD to a leg with or without an ulcer (clinical class 2–5 according to the CEAP classification; **Table** **1**), undergoing surgery. Surgical specimens of 3–9 mm of length were processed for *in situ* morphological analysis and/or endothelial cell culture isolation based on the sample size. As shown in **Figure** **1**, at morphological analysis, performed by SEM, normal vein (Control) showed a virtually intact endothelial layer. This appearance changed completely in disease specimens that displayed a progressive loss of the integrity of the endothelial monolayer as evidenced by craters or cavities and partially detached cells. At higher magnifications we could observed the presence of plaques covering the endothelial surface, red cells sticking to the endothelium and/or presence of numerous microvilli covering the endothelial surface that might reflect increased permeability of dysfunctional endothelial cells (**Figure** **1**).

### Isolation and phenotypically characterization of large vein endothelial cells

**Figure 2:**
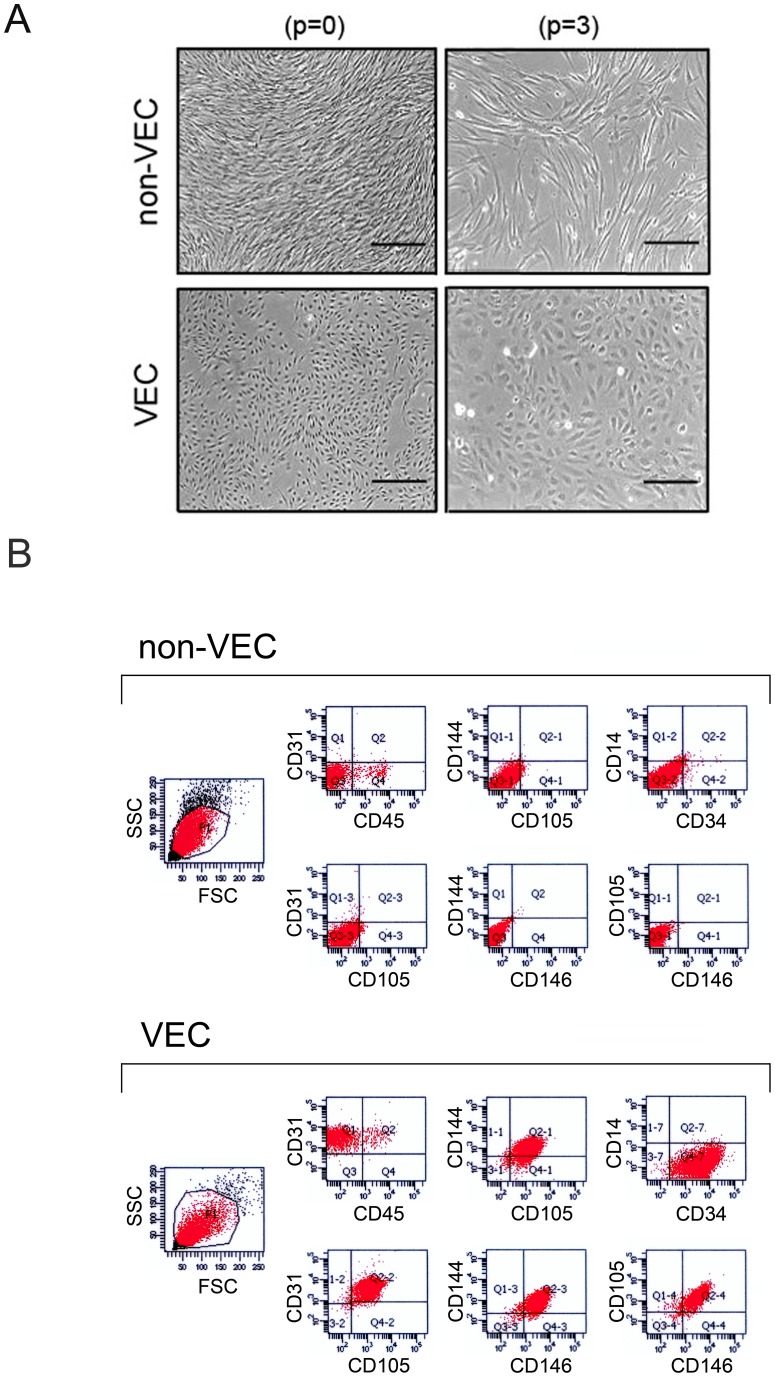
Isolation and phenotypic characterization of large vein endothelial cells. Cells isolated from surgical specimens were characterized *in-vitro* by morphological assessment (**A**) and multiparametric flow cytometry analysis (**B**). In **A**, cell morphology was examined by using phase-contrast microscopy. VEC cultures were characterized by regular polygonal shape and dimensions and uniform monolayer. On the other hand, non-VEC cultures appeared with a fibroblast-like morphology characterized by elongated shapes and growing in an uneven manner. Representative images of VEC and non-VEC cultures, at two different *in vitro* passages (p = 0 and p = 3), are shown. Left panels: 10X, original magnification; right panels: 20X original magnifications. In **B**, multiparametric flow cytometry analyses were performed with a specific panel of endothelial cells defining antibodies. VEC were defined as CD146^+^/CD144^+^/CD31^+^/CD105^+^/CD34^+^/CD45^−^/CD14^−^ while non-VEC displayed a more variable and random pattern of antigens expression. Two representative multiparametric flow cytometry analysis panels of a non-VEC and pure VEC cultures are shown as two-colors dot plots.

Among the 54 surgical specimens, the quality and the size of the specimens have allowed endothelial cell isolation in 47 out of 54 specimens. The morphology of the cultures has been examined regularly during the expansion process in order to identify the nature of the isolated cells and to confirm the healthy status of the cultures. Typically, one week after seeding it was already possible to distinguish between predominantly VEC and non-VEC cultures. As shown in **Figure** **2A**, VEC were polygonal in shape with regular dimensions; they grew uniformly attached to the substrate and the potential contamination of other cell types (presumably stromal cells) that characterized the first days of culture normally disappeared within few *in vitro* passages due to the predominance of endothelial cells. On the contrary, non-VEC were normally fibroblastic (or fibroblast-like) cells, bipolar or multipolar, showing elongated shapes and growing attached in either uniform way or distinct clusters (**Figure** **2A**). In parallel, a multicolor flow cytometry protocol was used to characterize VEC cultures based on the exclusion of cells expressing CD45 (a hematopoietic marker) and CD14 (expressed mainly by macrophages). VEC cultures were positive for CD146, CD144, CD31, CD105 and CD34 and, as far as the detection of contaminant hematopoietic cells within the endothelial monolayers, the low levels of CD45^+^ cells observed in cultures at first passage tended to decrease during the *in vitro* expansion (**Figure** **2B**). The non-VEC cultures showed a more variable immune-phenotype going from a complete negativity for the expression of the selected markers (**Figure** **2B**), to random levels of expression for single antigens. No relationship was observed between the age of patients and the efficiency of VEC isolation. Once we have defined the isolated cells as VEC (based on the multiparametric phenotypic characterization), cultures were expanded and after two-three passages, cells were further characterized for culture purity. As result, 21 VEC cultures obtained from patients affected by CVD (8 C2 and 13 C3) were selected on the base of purity>90%, as defined by CD31^+^/CD105^+^/CD146^+^/CD144^+^/CD45^−^/CD14^−^, and used for the subsequent *in vitro* functional and molecular investigations. CD34 marker was not used in this stage of VEC culture characterization since it tends to be variably lost during the culture passages.

### VEC exhibit differential phenotype and *in vitro* migratory and proliferation activities

**Figure 3:**
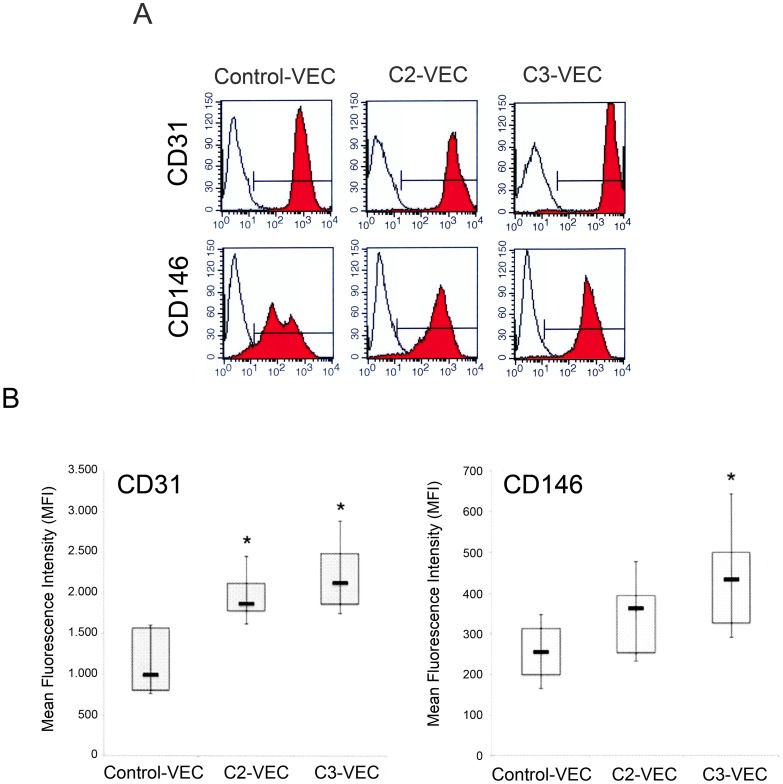
Differential phenotype between control and pathological VEC. Surface expression of CD31 and CD146 was evaluated by flow cytometry in either pathological and control-VEC. In **A**, colored histograms represent cells stained with monoclonal antibodies specific for the indicated antigens and white histograms represent background fluorescence obtained by staining the same cells with isotype-matched control antibodies. Representative panels for control, C2- and C3-VEC are shown. In **B**, the expression levels of the indicated antigens were determined for all VEC samples (8 C2-VEC, 13 C3-VEC and 5 control-VEC) by flow cytometry analysis and expressed as mean fluorescence intensity (MFI). Horizontal bars are median, upper and lower edges of box are 75th and 25th percentiles, lines extending from box are 10th and 90th percentiles. *P<0.05 compared to control VEC.

**Figure 4:**
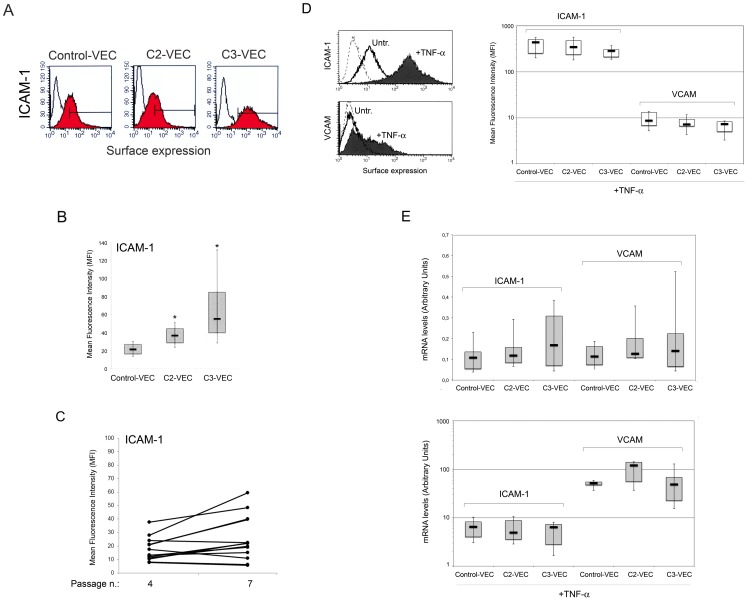
Analysis of ICAM-1 and VCAM expression in control and pathological VEC. In **A–C**, surface expression of ICAM-1 antigen was evaluated by flow cytometry in either pathological and control-VEC. In **A**, colored histograms represent cells stained with monoclonal antibodies specific for the indicated antigens and white histograms represent background fluorescence obtained by staining the same cells with isotype-matched control antibodies. A representative panel for control-, C2- and C3-VEC is shown. In **B**, the expression levels of ICAM-1 were determined for all VEC samples (8 C2-VEC, 13 C3-VEC and 5 control VEC) by flow cytometry analysis and expressed as mean fluorescence intensity (MFI). *P<0.05 compared to control VEC. In **C**, comparative analysis of ICAM-1 surface expression, reported as MFI, at different VEC passages. In **D–E**, pathological and control-VEC cultures were exposed to TNF-α for 18 hours before ICAM-1 and VCAM surface expression analysis by flow cytometry (**D**), and mRNA levels analysis by quantitative RT-PCR (**E**). In **D**, two representative panels are shown: dotted histograms represent background fluorescence obtained by staining the same cells with isotype-matched control antibodies. The expression levels of ICAM-1 and VCAM, determined for all VEC samples by flow cytometry analysis upon TNF-α stimulation, are expressed as mean fluorescence intensity (MFI). In **E**, mRNA expression levels of ICAM-1 and VCAM were determined both in unstimulated and TNF-α-stimulated VEC cultures. Results from amplifications, done in duplicate, are expressed as arbitrary units, after normalization for the housekeeping gene. Horizontal bars are median, upper and lower edges of box are 75th and 25th percentiles, lines extending from box are 10th and 90th percentiles.

At the time of the *in vitro* functional assays, pathological VEC (n = 21) and control VEC cultures (n = 5) were characterized for the surface expression of the following molecular markers: CD146, CD31, CD105, VCAM, ICAM-1. Even though the percentage of positive cells expressing CD31 and CD146 was >90% in all VEC cultures, pathological VEC showed a significantly (p<0.05) increased expression of CD31 and CD146 compared to control VEC with a growing trend in C3-VEC as compared to C2-VEC (**Figure** **3A–B**). On the other hand, CD105 was highly expressed (>90%) in all VEC cultures without showing significant differences among pathological and normal cultures (data not shown). In addition, also the surface expression level of ICAM-1, analysed by flow-cytometry (**Figure** **4A–C**), was significantly (p<0.05) higher in C3-VEC compared to C2-VEC and normal-VEC. On the other hand, VCAM was undetectable at the surface levels in unstimulated cultures, although similarly to ICAM-1, its expression was significantly induced both at protein (**Figure** **4D**) and mRNA (**Figure** **4E**) level in response to TNF-α.

**Figure 5:**
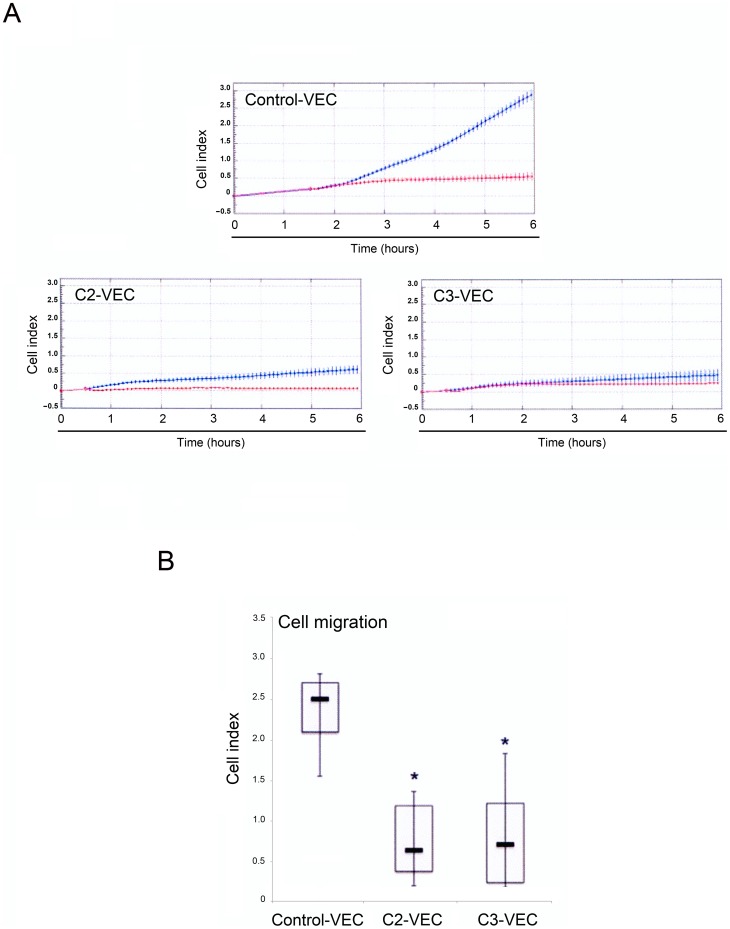
Differential migration kinetics between control and pathological VEC. Control and pathological VEC cells were seeded at 2.5x10^4^ cells in fibronectin-coated 16 wells CIM-plates, and migration kinetics were analyzed in the absence or presence of 10% FBS in the bottom chamber and recorded by the xCELLigence real time cell analyzer. Cell migration was evaluated for all VEC cultures by using the recording changes in impedance and was expressed as cell index (CI). In **A**, representative panels of control-VEC, C2-VEC and C3-VEC migration, expressed as CI mean±SD (with samples assayed in quadruplicate), are shown. Blue lines: specific migration through FBS gradient; red lines, background migration. In **B**, horizontal bars are median, upper and lower edges of box are 75th and 25th percentiles, lines extending from box are 10th and 90th percentiles. *P<0.05 compared to control VEC.

We next analysed biological key functions of endothelial cells: the migratory and the proliferative/survival capabilities. The directed migration response of VEC toward serum (10% FCS) was investigated using the real-time and label-free monitoring of cell migration based on electrical impedance real-time cell analysis (RTCA). As shown in **Figure** **5A–B**, the migration of C3- and C2-VEC, recorded 6 hours after cell plating, was comparable in terms of cell index. On the other hand, control VEC showed a significantly (p<0.05) higher migration ability compared to pathological VEC (**Figure** **5B**).

**Figure 6:**
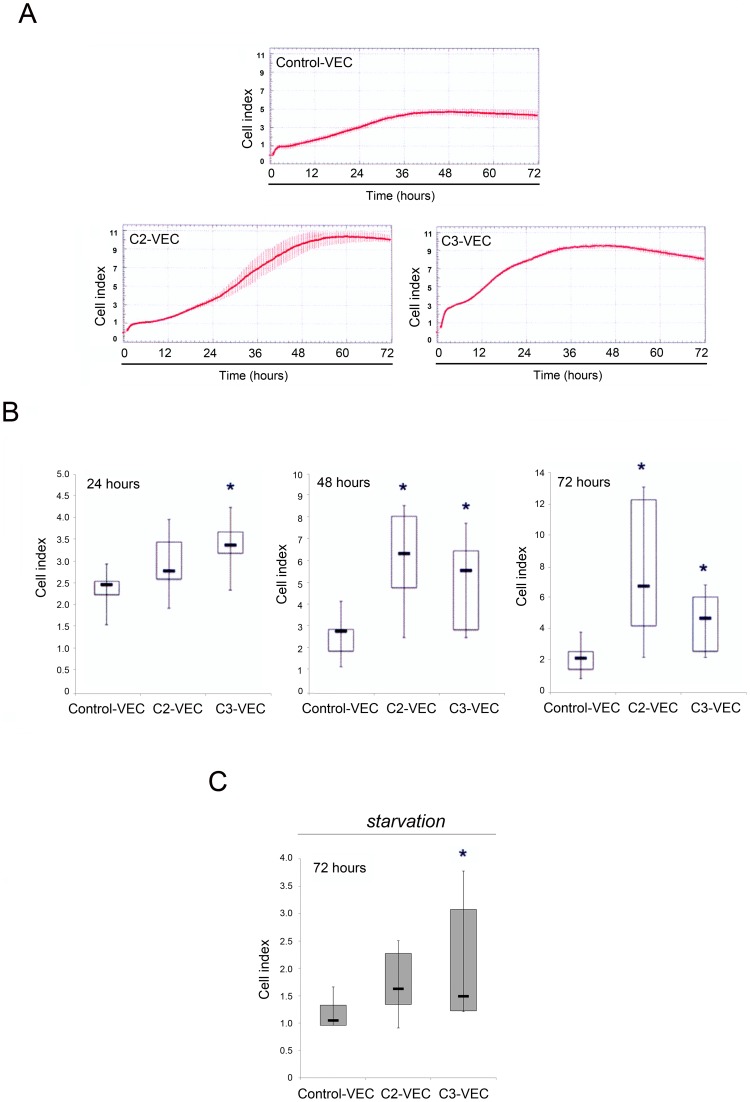
Differential proliferation kinetics between control and pathological VEC. Control and pathological VEC cells were seeded in fibronectin pre-coated 16 wells E-plates and monitored using the xCELLigence real time cell analyzer (RTCA). In **A–B**, cell proliferation was evaluated for all VEC samples and was expressed as cell index (CI) after normalization to the CI recorded at 4 hours. In **A**, representative panels of control-VEC, C2-VEC and C3-VEC proliferation, expressed as CI mean±SD (with samples assayed in quadruplicate), are shown. In **B**, horizontal bars are median, upper and lower edges of box are 75th and 25th percentiles, lines extending from box are 10th and 90th percentiles. *P<0.05 compared to control VEC. In C, data were generated by monitoring cell index in conditions of starvation (72 hours of serum and growth factor deprivation). *P<0.05 compared to control VEC.

In contrast to the migration data, pathological VEC showed a significantly (p<0.05) higher spontaneous proliferation activity when cultured in complete (supplemented with serum and growth factors) medium, anticipated in C3-VEC (24 hours), with respect to control-VEC (**Figure** **6A–B**). Even in starvation conditions (72 hours in the absence of serum and growth factors), the cell index of C3-VEC was significantly (p<0.05) higher than control VEC, indicating a higher resistance to growth factors deprivation (**Figure** **6C**).

### C3-VEC are characterized by higher levels of NF-kB activity and by increased release of the pro-inflammatory cytokines OPG and VEGF

**Figure 7:**
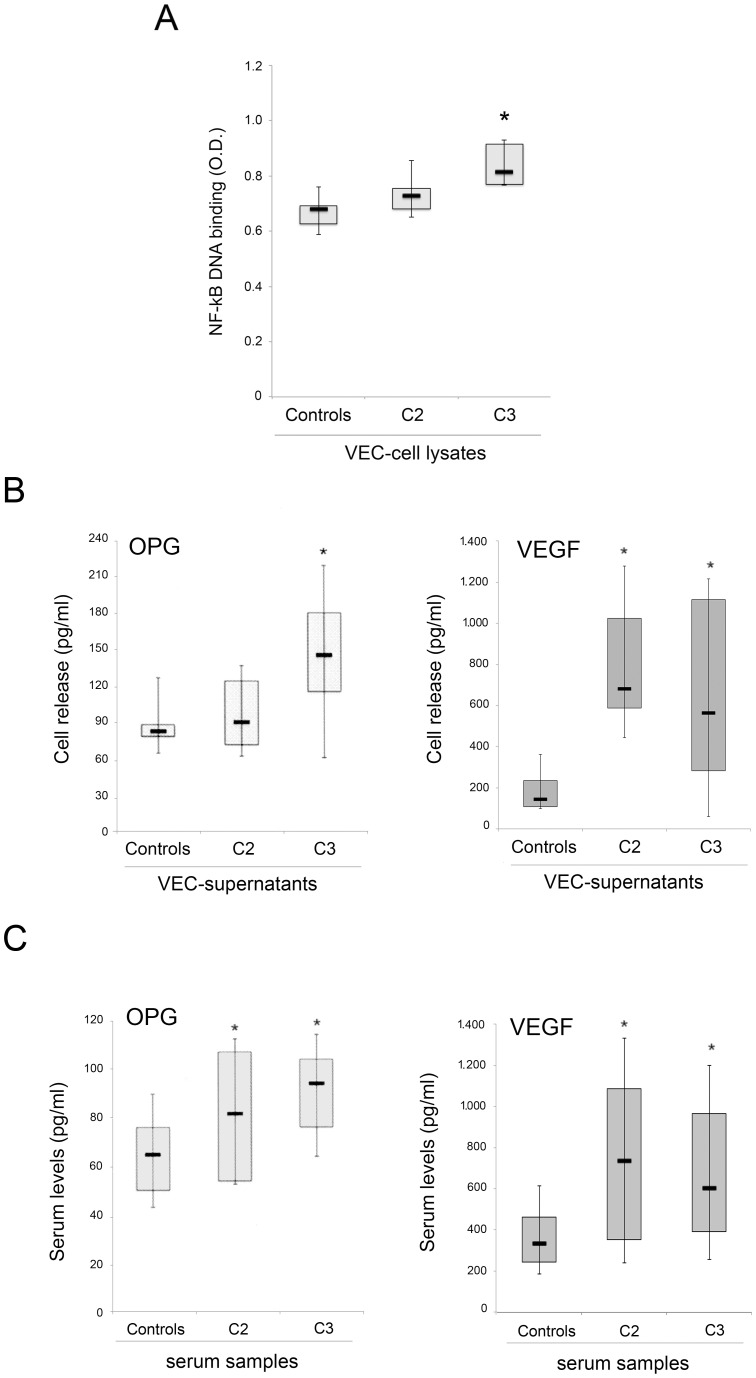
Analysis of the NF-kB, OPG and VEGF levels in VEC culture supernatants and serum samples. In **A**, NF-kB-p65 DNA binding activity was assessed in duplicate using the TransAM assay. Results are reported as absorbance values (O.D.) per 20 µg of cell lysate protein. In **B**, OPG and VEGF levels were determined by ELISA in VEC culture supernatants. In **C**, OPG and VEGF levels were determined by ELISA in sera of C2 and C3 patients as well as in sex and age-matched normal controls. In **A–C**, horizontal bars are median, upper, and lower edges of box are 75th and 25th percentiles; lines extending from box are 10th and 90th percentiles. *, *P*<0.05 compared to either control VEC (**A–B**) or control sera (**C**).

In order to start to dissect the molecular mechanism underlining the pro-inflammatory phenotype of pathological VEC, we assessed the activation levels of NF-kB, by analysing VEC cell lysates for binding activity of p65/RelA to an oligonucleotide containing the *k*B consensus site, in TransAM assays (**Figure** **7A**). Of note, a significant (p<0.05) higher baseline NF-kB activity was measured in C3- with respect to C2- and control-VEC. Consistently, both OPG and VEGF, which are transcriptional targets of NF-kB [21], [22] and represent important mediators of endothelial inflammation and/or angiogenesis, were released at significantly (p<0.05) higher levels in the culture supernatant of pathological VEC with respect to control-VEC (**Figure** **7B**). Moreover, since both OPG and VEGF exert pro-survival and proliferative effects in endothelial cells, it is likely that both cytokines contribute to the increased proliferation activity of C3-VEC observed in both complete medium and under starvation (**Figure** **6A–C**).

In the last group of experiments, we have analysed whether the elevation of OPG and VEGF observed in the culture supernatants of C3-VEC might have a systemic correlate. For this purpose, we have measured the serum levels of OPG and VEGF in a group of C2 and C3 patients as well as in sex and age-matched normal controls. As shown in **Figure** **7C**, CVD-patients showed significantly (p<0.05) higher levels of circulating OPG and VEGF with respect to normal controls.

## Discussion

How the inflammatory properties of vein endothelium stand in relation to CVD was completely unknown. Therefore, we reasoned that data elucidating the characteristics of large vein endothelium would increase our knowledge of large vessel physiopathology. By analysing 21 pure pathological VEC cultures, derived from C2 and C3-stage of disease according to the CEAP classification, in comparison with 5 normal VEC, we were able to demonstrate that pathological VEC showed distinct *in vitro* characteristics with respect to normal VEC: i) increased surface expression of CD146, CD31/PECAM-1 and ICAM-1; ii) increased cell proliferation activity; iii) increased survival in starvation conditions, iv) decreased spontaneous migration; v) increased release of OPG and VEGF. Importantly, at molecular levels, pathological VEC were characterized by an increased baseline transcriptional activity of NF-kB with respect to control VEC. Moreover, the increased *in vitro* release of OPG and VEGF by pathologic VEC showed a systemic correlate represented by elevated serum levels of OPG and VEGF in C3 and C2 patients with respect to sex- and age-matched controls.

It is noteworthy that the surface markers significantly up-regulated in pathological versus normal VEC (CD146, CD31/PECAM-1 and ICAM-1) have been involved in different aspects of the inflammatory response of endothelial cells. In particular, CD146 and its soluble form regulate monocyte trans-endothelial migration [23] and promote angiogenesis [24]. CD31/PECAM-1 is vital to the regulation of inflammatory responses, as it has been shown to serve a variety of pro-inflammatory functions, such as the facilitation of leukocyte trans-endothelial migration and the transduction of mechanical signals in endothelial cells emanating from fluid shear stress [24]–[26]. However, CD31/PECAM-1 has also been involved in the maintenance of vascular barrier integrity. Finally, ICAM-1 is known to promote recruitment of leukocytes, platelets, and erythrocytes to the vein wall [27], [28].

Taken together, these data are suggestive of a pronounced pro-inflammatory profile in CVD endothelial cells, which correlates with the disease stage and differs from normal venous endothelial cells. In keeping with this hypothesis, the enhanced release of OPG by C3-VEC is an event coherent with a more advanced inflammatory condition in C3>C2>normal endothelial cells. In fact, we have previously demonstrated that part of the pro-inflammatory activity of OPG is due to its ability to act as a bridge between leukocytes and endothelial cells [16]. Our current findings are in line with previous studies indicating that specific inflammatory mediators are associated with time- and pattern-dependent healing progression in CVD [29]. In addition, it has been shown that endothelial dysfunction is present in varicose veins and that this anomaly can be reverted by cardiovascular protecting agents [30]. In the context of our study, it is noteworthy that an association has been described between symptoms of CVD in the lower extremities and cardiovascular risk factors [31]–[33]. In particular, in one study, after excluding the effect of age, CVD symptoms were still strongly associated with a history of coronary artery disease and a cluster of cardiovascular risk factors that included higher values of body mass index, waist circumference and serum triglycerides [33]. These results support the hypothesis that there is an overlap between atherosclerotic and venous disorders. In this respect, it is noteworthy that several studies have previously demonstrated that elevated circulating levels of OPG, as well as of VEGF, represent a risk factor for cardiovascular disease [34]–[38].

In conclusion, we have established that endothelial cells obtained from large veins affected by an intermediate degree of CVD display a shift towards a pro-inflammatory phenotype. Of note, several pro-inflammatory markers (CD31, CD146, ICAM-1 and OPG) showed a gradient from normal to pathological VEC, which was related to the stage (C2 or C3) of the disease. In addition, the soluble inflammatory markers OPG and VEGF were increased not only in the culture supernatant, but also in the general circulation, suggesting that VEC significantly contribute to promote systemic inflammation.

## References

[1] Eberhardt RT, Raffetto JD (2005). Chronic venous insufficiency.. Circulation.

[2] Parkington HC, Coleman HA, Tare M (2004). Prostacyclin and endothelium dependent hyperpolarization.. Pharmacol Res.

[3] Levi M, Keller TT, van Gorp E, ten Cate H (2003). Infection and inflammation and the coagulation system.. Cardiovasc Res.

[4] von Andrian UH, Mackay CR (2000). T-Cell function and migration. Two sides of the same coin.. N Engl J Med.

[5] Ulbrich H, Eriksson EE, Lindbom L (2003). Leukocyte and endothelial cell adhesion molecules as targets for therapeutic interventions in inflammatory disease.. Trends Pharmacol Sci.

[6] Lusis AJ (2000). Atherosclerosis.. Nature.

[7] Eriksson EE (2003). Leukocyte recruitment to atherosclerotic lesions, a complex web of dynamic cellular and molecular interactions.. Curr Drug Targets Cardiovasc Haematol Disord.

[8] Wakefield TW, Strieter RM, Prince MR, Downing LJ, Greenfield LJ (1997). Pathogenesis of venous thrombosis: a new insight.. Cardiovasc Surg.

[9] Gimbrone MA, Topper JN, Nagel T, Anderson KR, Garcia-Cardena G (2000). Endothelial dysfunction, hemodynamic forces, and atherogenesis.. Ann N Y Acad Sci.

[10] Ley K (2002). Integration of inflammatory signals by rolling neutrophils.. Immunol Rev.

[11] Sumpio BE, Riley JT, Dardik A (2002). Cells in focus: endothelial cell.. Int J Biochem Cell Biol.

[12] Lawson ND, Weinstein BM (2002). Arteries and veins: making a difference with zebrafish.. Nat Rev Genet.

[13] Eriksson EE, Karlof E, Ludmark K, Rotzius P, Hedin U (2005). Powerful inflammatory properties of large vein endothelium in vivo.. Arterioscler Thromb Vasc Biol.

[14] Schmid-Schonbein GW, Takase S, Bergan JJ (2001). New advances in the understanding of the pathophysiology of chronic venous insufficiency.. Angiology.

[15] Zauli G, Melloni E, Capitani S, Secchiero P (2009). Role of full-length osteoprotegerin in tumor cell biology.. Cell Mol Life Sci.

[16] Zauli G, Corallini F, Bossi F, Fischietti F, Durigotto P (2007). Osteoprotegerin increases leukocyte adhesion to endothelial cells both in vitro and in vivo.. Blood.

[17] Campioni D, Corallini A, Zauli G, Possati L, Altavilla G (1995). HIV type 1 extracellular tat protein stimulates growth and protects cells of BK virus/tat transgenic mice from apoptosis.. AIDS Res Hu Retrov.

[18] Secchiero P, Zerbinati C, di Iasio MG, Melloni E, Tiribelli M (2007). Synergistic cytotoxic activity of recombinant TRAIL plus the non-genotoxic activator of the p53 pathway nutlin-3 in acute myeloid leukemia cells.. Curr Drug Metab.

[19] Voltan R, di Iasio MG, Bosco R, Valeri N, Pekarski Y (2011). Nutlin-3 downregulates the expression of the oncogene TCL1 in primary B chronic lymphocytic leukemic cells.. Clin Cancer Res.

[20] Secchiero P, Corallini F, Beltrami AP, Ceconi C, Bonasia V (2010). An imbalanced OPG/TRAIL ratio is associated to severe acute myocardial infarction.. Atherosclerosis.

[21] Malyankar UM, Scatena M, Suchland KL, Yun TJ, Clark EA (2000). Osteoprotegerin is an alpha vbeta 3-induced, NF-kappa B-dependent survival factor for endothelial cells.. J Biol Chem.

[22] Leychenko A, Konorev E, Jijma M, Matter ML (2011). Strech-induced hypertrophy activates NF-kB-mediated VEGF secretion in adult cardiomyocytes.. PloS One.

[23] Bardin N, Blot-Chabaud M, Despoix N, Kebir A, Harhouri K (2009). CD146 and its soluble form regulate monocyte transendothelial migration.. Arterioscler Thromb Vasc Biol.

[24] Harhouri K, Kebir A, Guillet B, Foucault-Bertaud A, Voytenko S (2010). Soluble CD146 displays angiogenic properties and promotes neovascularization in experimental hind-limb ischemia.. Blood.

[25] Glen K, Luu NT, Ross E, Buckley CD, Rainger GE (2012). Modulation of functional responses of endothelial cells linked to angiogenesis and inflammation by shear stress: differential effects of the mechanotransducer CD31.. J Cell Physiol.

[26] Privratsky JR, Newman DK, Newman PJ (2010). PECAM-1: conflicts of interest in inflammation.. Life Sci.

[27] Lawson C, Wolf S (2009). ICAM-1 signaling in endothelial cells.. Pharmacol Rep.

[28] Alcaide P, Maganto-Garcia E, Newton G, Travers R, Croce KJ (2012). Difference in Th1 and Th17 lymphocyte adhesion to endothelium.. J Immunol.

[29] Beidler SK, Douillet CD, Berndt DF, Keagy BA, Rich PB (2009). Inflammatory cytokine levels in chronic venous insufficiency ulcer tissue before and after compression therapy.. J Vasc Surg.

[30] Carrasco OF, Ranero A, Hong E, Vidrio H (2010). Endothelial function impairment in chronic venous insufficiency: effect of some cardiovascular protectant agents.. Angiology.

[31] Prandoni P, Bilora F, Marchiori A, Bernardi E, Petrobelli F (2003). An association between atherosclerosis and venous thrombosis.. N Engl J Med.

[32] Hong C, Zhu F, Du D, Pilgram TK, Sicard GA (2005). Coronary artery calcification and risk factors for atherosclerosis in patients with venous thromboembolism.. Atherosclerosis.

[33] Auzky O, Lanska V, Pitha J, Roztocil K (2011). Association between symptoms of chronic venous disease in the lower extremities and cardiovascular risk factors in middle-aged women.. Int Angiol.

[34] Venuraju SM, Yerramasu A, Corder R, Lahiri A (2010). Osteoprotegerin as a predictor of coronary artery disease and cardiovascular mortality and morbidity.. J Am Coll Cardiol.

[35] Vik A, Mathiesen EB, Brox J, Wilsgaard T, Njølstad I (2011). Serum osteoprotegerin is a predictor for incident cardiovascular disease and mortality in a general population: the Tromsø Study.. J Thromb Haemost.

[36] Mogelvang R, Pedersen SH, Flyvbjerg A, Bjerre M, Iversen AZ (2012). Comparison of osteoprotegerin to traditional atherosclerotic risk factors and high-sensitivity C-reactive protein for diagnosis of atherosclerosis.. Am J Cardiol.

[37] Westra J, de Groot L, Plaxton SL, Brouwer E, Posthumus MD (2011). Angiopoietin-2 is highly correlated with inflammation and disease activity in recent-onset rheumatoid arthritis and could be predictive for cardiovascular disease.. Rheumatology.

[38] Naskret D, Zozulinska-Ziolkiewicz DA, Dankowski R, Wysocki H, Wierusz-Wysocka B (2010). Albuminuria and VEGF as early markers of cardiovascular disturbances in young type 1 diabetic patients.. Microvasc Res.

